# Endocytosis of somatodendritic NCKX2 is regulated by Src family kinase-dependent tyrosine phosphorylation

**DOI:** 10.3389/fncel.2013.00014

**Published:** 2013-02-20

**Authors:** Kyu-Hee Lee, Won-Kyung Ho, Suk-Ho Lee

**Affiliations:** Department of Physiology, Biomembrane Plasticity Research Center and Neuroscience Research Institute, Seoul National University College of MedicineSeoul, Republic of Korea

**Keywords:** NCKX2, AP-2, tyrosine phosphorylation, endocytosis, Src family kinase, dendrite, calcium clearance

## Abstract

We have previously reported that the surface expression of K^+^-dependent Na^+^/Ca^2+^ exchanger 2 (NCKX2) in the somatodendritic compartment is kept low by constitutive endocytosis, which results in the polarization of surface NCKX2 to the axon. Clathrin-mediated endocytosis is initiated by interaction of the μ subunit of adaptor protein complex 2 (AP-2) with the canonical tyrosine motif (YxxΦ) of a target molecule. We examined whether endocytosis of NCKX2 involves two putative tyrosine motifs (^365^YGKL and ^371^YDTM) in the cytoplasmic loop of NCKX2. Coimmunoprecipitation assay revealed that the ^365^YGKL motif is essential for the interaction with the μ subunit of AP-2 (AP2M1). Consistently, either overexpression of NCKX2-Y365A mutant or knockdown of AP2M1 in cultured hippocampal neurons significantly reduced the internalization of NCKX2 from the somatodendritic surface and thus abolished the axonal polarization of surface NCKX2. Next, we tested whether the interaction between the tyrosine motif and AP2M1 is regulated by phosphorylation of the 365th tyrosine residue (Tyr-365). Tyrosine phosphorylation of heterologously expressed NCKX2-WT, but not NCKX2-Y365A, was increased by carbachol (CCh) in PC-12 cells. The effect of CCh was inhibited by PP2, a Src family kinase (SFK) inhibitor. Moreover, PP2 facilitated the endocytosis of NCKX2 in both the somatodendritic and axonal compartments, suggesting that tyrosine phosphorylation of NCKX2 by SFK negatively regulates its endocytosis. Supporting this idea, activation of SFK enhanced the NCKX activity in the proximal dendrites of dentate granule cells (GCs). These results suggest that endocytosis of somatodendritic NCKX2 is regulated by SFK-dependent phosphorylation of Tyr-365.

## Introduction

Calcium is the most versatile second messenger in cell signal transduction. Dendrite calcium signaling can modulate a wide range of dendritic functions such as surface expression of ion channels (Kim et al., 2007), reorganization of cytoskeleton (Liu et al., 2008), and gene expression (Lee et al., 2011), which lead to synaptic plasticity. Despite the versatile nature of calcium, it is not promiscuous, because the spatial and temporal extents of calcium signaling are highly regulated by calcium influx, cytosolic calcium buffers and calcium clearance mechanisms (CCMs). Such regulation mechanisms may play an essential role in specifying the downstream coupling of a calcium signal.

The plasma membrane Ca^2+^-ATPase (PMCA) and Na^+^/Ca^2+^ exchanger (NCX) have been known to be major CCMs in dendrites. PMCA has higher affinity for calcium but is easily saturated at high [Ca^2+^] (Kim et al., 2003; Lee et al., 2009). Because NCX shows higher turnover rate and capacity than PMCA, NCX has been considered a major CCM at high [Ca^2+^] in dendrites. Dendritic NCX is a key player in the modulation of the spatial extent of calcium signals induced by excitatory synaptic inputs (Goldberg et al., 2003; Lörincz et al., 2007). Accordingly, deletion of genes encoding different forms of NCX causes alterations in synaptic plasticity and cognitive functions (Jeon et al., 2003; Molinaro et al., 2011).

In contrast to NCX, dendritic function of the K^+^-dependent Na^+^/Ca^2+^ exchanger (NCKX) is controversial. Previously, based on studies of the decay phase of calcium transients, we have reported that NCKX activity is the major CCM at axon terminals but is not detectable in somatodendritic regions of central neurons (Kim et al., 2003, 2005; Lee et al., 2009). Furthermore, we have shown that the surface expression of NCKX2 is polarized to the axonal compartment (Lee et al., 2012) in cultured hippocampal neurons. This view is in disagreement with other studies, which showed the direct recording of NCKX current or Ca^2+^-influx via the reverse mode of NCKX in the somata of cortical neurons (Kiedrowski et al., 2004; Cuomo et al., 2008). Indeed, we have previously reported that not only axon terminals but also somatodendritic regions are immunoreactive for NCKX2 in cultured hippocampal neurons under permeabilized conditions. Furthermore, suppression of endocytosis allowed the dendritic surface expression of NCKX2, suggesting that constitutive endocytosis of NCKX2 from somatodendritic compartments contributes to the axonal polarization of the NCKX2 activity (Lee et al., 2012). These previous findings prompted us to study the molecular mechanism regulating the surface expression of NCKX2 in dendrites of central neurons.

Clathrin-mediated endocytosis is the major pathway for internalization of plasma membrane proteins, and is initiated by recruitment of the adaptor protein complex, AP-2, to the target molecule. The AP-2 complex is composed of four subunits: two large (α, β), medium (μ) and small (σ) subunits (Kirchhausen, 1999). Among endocytotic adaptors, the AP-2 complex plays a central role in the endocytosis of plasma membrane proteins, since the μ subunit of AP-2 displays a high degree of specificity for phosphatidylinositol-4,5-bisphosphate, which is enriched in the plasma membrane (Rohde et al., 2002). AP-2 recognizes canonical or non-canonical endocytosis motif of a target molecule. There are two well-known canonical endocytosis motifs. One is a dileucine motif ([D/E]xxxL[L/I]; x = any amino acid) recognized by the β subunit of AP-2 (Rapoport et al., 1998), and the other is a tyrosine motif (YxxΦ; Φ = hydrophobic amino acid) recognized by the μ subunit of AP-2 (AP2M1) (Ohno et al., 1995). NCKX2 has two tyrosine motifs (^365^YGKL and ^371^YDTM) in its intracellular loop region (Tsoi et al., 1998). Surface expression of many ion channels and neurotransmitter receptors are regulated by phosphorylation of tyrosine motifs (Ohnishi et al., 2011), because phosphorylated tyrosine motif renders it resistant to AP-2-mediated endocytosis.

We tested whether the tyrosine motifs of NCKX2 are involved in the endocytic regulation of its dendritic surface expression. Here, we report that the first tyrosine motif of NCKX2 interacts with AP2M1 and that the interaction is required for endocytosis of NCKX2 from the dendritic surface. Furthermore, we show that a Src family kinase (SFK) modulates the endocytosis of NCKX2 by tyrosine-phosphorylation of the AP-2 recognition motif in NCKX2.

## Materials and methods

### DNA constructs

The full length rat NCKX2 (NM_031743.2; O54701) was cloned into pcDNA3.1(+) plasmid (Invitrogen, Carlsbad, CA, USA). The cytoplasmic loop region of NCKX2 [NCKX2-loop, amino acids (aa) 288–478] was isolated from full length NCKX2 and subcloned into the mammalian expression vector pcDNA3.1(+) with N-terminal c-myc tag (myc-NCKX2-loop). The NCKX2-FLAG construct was created by replacing the N-terminal 8 amino acids (aa 90–97; DLNDKIRD) by the FLAG tag (DYKDDDDK). Y365A and/or Y371A or Y365E mutants were obtained by using QuickChange Site-Directed Mutagenesis kit (Agilent Technologies, Palo Alto, CA, USA). The HA-tagged AP2M1 (HA-AP2M1) (NM_053837.1; P84092) cloned into pcDNA3 and non-targeting shRNA (sh-NT) or AP2M1-targeting shRNA (sh-AP2M1) cloned into pSuper (OligoEngine, Seattle, WA, USA) were gifts from Dr. Ryan (Weill Cornell Medical College) (Kim and Ryan, 2009). The luciferase-targeting siRNA sequence (5′-TAAGGCTATGAAGAGATAC-3′) was used as a non-targeting siRNA control (Dharmacon, Lafayette, CO, USA). tdTomato was used for visualizing the entire morphology of a cultured hippocampal neuron.

### Cell culture

Hippocampal neurons were cultured on a coverslip suspended above an astrocyte feeder layer. Hippocampal neurons and glia were obtained from Sprague-Dawley rats according to the protocols approved by the Seoul National University Institutional Animal Care and Use Committee. The protocol for low-density neuron-glia co-culture has been previously described (Kaech and Banker, 2006). Two weeks before neuronal culture, astrocytes were obtained by passing a cortical cell suspension from postnatal day (P) 1 rat through a cell strainer (40 μm mesh, BD Falcon, Franklin Lakes, NJ, USA), and then cultured in glial medium [minimum essential medium (MEM; Invitrogen) supplemented with 0.6% glucose, 1 mM pyruvate, 2 mM GlutaMAX-I (Invitrogen), 10% horse serum (HS; Invitrogen), and 1% penicillin-streptomycin (PS; Invitrogen)]. Hippocampi from embryonic day (E) 18 fetal rats were dissected in Hank's balanced salt solution (Invitrogen), digested with papain (Worthington, Freehold, NJ, USA), and then triturated with a polished half-bore Pasteur pipette. The neurons in the plating medium [the same composition with the glial medium except for 10% fetal bovine serum (FBS; Invitrogen) instead of HS] were plated on a poly-D-lysine (Sigma, St. Louis, MO, USA)-coated glass coverslip (Marienfeld, Lauda-Königshofen, Germany) in a 60-mm culture dish at a density of 0.7–1.4 × 10^4^ cells/cm^2^. Paraffin dots (“feet” to suspend the coverslips above the glial feeder layer) were applied to the coverslips before neuron plating. The next day, coverslips were transferred above the glial culture pre-incubated in Neurobasal A medium (Invitrogen) supplemented with 0.5 mM GlutaMAX-I and 2% B-27 supplement (Invitrogen) for a day. To prevent proliferation of glial cells, 5 μM of 1-β-D-cytosine-arabinofuranoside (Sigma) was added at the 4th day *in vitro* (DIV4).

HEK293 cells (ATCC) were plated at a density of 5 × 10^4^ cells per 100-mm culture dishes and maintained in the Dulbecco's modified Eagle's medium (Invitrogen) supplemented with 10% FBS and 1% PS. PC-12 cells (Korean Cell Line Bank, Seoul, South Korea) were plated at a density of 5 × 10^4^ cells per a 0.01% poly-L-lysine (Sigma)-coated 100-mm culture dish, and maintained in the RPMI-1640 medium (Invitrogen) supplemented with 10% HS, 5% FBS, and 1% PS.

### Transfection

Primary hippocampal neurons (DIV7-8) were transfected using calcium phosphate (Ryan et al., 2005). Before transfection, the culture medium was replaced by 2 ml of Neurobasal A containing 25 mM HEPES (pH 7.3, adjusted with NaOH) with the conditioned culture medium saved. The DNA/calcium phosphate precipitate was prepared by mixing one volume of DNA (up to 10 μg) in 250 mM CaCl_2_ with an equal volume of 2× HBS (280 mM NaCl, 50 mM HEPES, 1.5 mM Na_2_HPO_4_, pH 7.1) using a vortex mixer. Then 200 μl DNA/calcium phosphate mixture was added dropwise to the cultured neurons, and neurons were incubated at 37°C for 15 min. After the incubation, DNA/calcium phosphate precipitates were washed out three times with fresh Neurobasal A for 5 min and the cells were returned to the saved original medium. For Figure 2, wild-type (WT) or Y365A mutant of NCKX2-FLAG and tdTomato were transfected to hippocampal neurons. For Figure 3, WT of NCKX2-FLAG and sh-NT or sh-AP2M1 were co-transfected in a ratio of 1:1. HEK293 cells were also transfected using calcium phosphate. The procedures were essentially the same except the medium was not changed before and after adding of DNA/calcium phosphate mixture to the culture. PC-12 cells were transfected with WT or Y365A mutant of NCKX2-FLAG using Lipofectamine 2000 (Invitrogen) according to the manufacturer's instructions.

### Coimmunoprecipitation and peptide elution

HEK293 cells were seeded in a 100-mm culture dish at approximately 70% confluence and transfected with HA-AP2M1 and the WT or mutant form (Y365A or Y371A) of myc-NCKX2-loop, using the calcium phosphate method. After culture for 20–48 h, the cells were washed twice with phosphate-buffered saline (PBS) and solubilized in ice-cold lysis buffer containing 50 mM Tris-Cl (pH 7.4), 150 mM NaCl, 1 mM EDTA, 0.5% Triton X-100 (v/v), and 1% protease inhibitor mixture. After incubation on ice for 30 min, cell lysates were clarified by centrifugation at 12,000× g for 10 min at 4°C. The supernatants containing 500 μg total protein were incubated with anti-c-myc antibody-conjugated agarose beads (Sigma) by gentle inverting overnight at 4°C. The beads were then washed three times with wash buffer containing 50 mM Tris-Cl (pH 7.4), 150 mM NaCl, 1 mM EDTA, and 0.1% Triton X-100 (v/v) for 10 min, and the bound proteins were eluted from the agarose beads by incubation with 500 μg/ml c-myc peptide (Sigma) diluted in lysis buffer for 30 min on ice. The immunoprecipitated protein complexes in the supernatant were denatured by boiling with 2× SDS sample buffer and subjected to SDS-PAGE and western blot analysis.

### Western blotting

To test the knockdown effect of sh-AP2M1, HEK293 cells expressing HA-AP2M1 and sh-NT or sh-AP2M1 were lysed using lysis buffer containing 50 mM Tris-Cl (pH 7.4), 150 mM NaCl, 1 mM EDTA, 1% SDS, and 1% protease inhibitor mixture. To detect phosphotyrosine (pTyr) residues of NCKX2, PC-12 cells were transfected with FLAG-tagged WT or Y365A mutant NCKX2. Two days later, we treated the transfected cells with 1 mM carbachol (CCh) or CCh plus 10 μM PP2 for 2 min (Calbiochem, San Diego, CA, USA). Immediately after the CCh treatment, we harvested the cell lysate using the same lysis buffer that we used for coimmunoprecipitation, with the addition of 1 mM Na_3_VO_4_. The cell lysates were pre-cleared by incubating with 25 μl of protein G-conjugated agarose beads (50% slurry, Santa Cruz Biotechnology, Santa Cruz, CA, USA). WT or Y365A mutant NCKX2 was immunoprecipitated from pre-cleared cell lysates of PC-12 cells using protein G agarose beads that had been pre-incubated with anti-FLAG.

Cell lysates or immunoprecipitated proteins were separated by SDS-PAGE and transferred onto a polyvinylidene difluoride membrane (Millipore, Billerica, MA, USA). The resulting blots were blocked for 1 h in PBS plus 0.1% Triton X-100 (0.1% PBST) containing 5% skim milk (Difco, Detroit, MI, USA). The blots were incubated overnight at 4°C with specific primary antibodies: mouse monoclonal anti-HA (1:1000, Covance, Princeton, NJ, USA), mouse monoclonal anti-c-myc (1:1000, Cell Signaling Technology, Beverly, MA, USA), rabbit polyclonal anti-FLAG (1:2000, Sigma), mouse monoclonal anti-pTyr (1:1000, Millipore) or mouse monoclonal anti-β-actin (1:5000, Santa Cruz Biotechnology) as a loading control. After washing three times with 0.1% PBST for 10 min, the blots were incubated at room temperature (RT) for 1 h with the corresponding horseradish peroxidase-conjugated secondary antibodies: goat anti-mouse IgG (1:5000, Jackson ImmunoResearch, West Grove, PA, USA) or goat anti-rabbit IgG (1:2000, Abcam, Cambridge, UK). After washing three times with 0.1% PBST for 10 min, detection was performed using enhanced chemiluminescence reagent (Amersham Bioscience, Buckinghamshire, UK). The membranes were then exposed to X-ray films (Agfa-Gevaert, Mortsel, Belgium). Films were digitally scanned and signals were quantified using the densitometric analysis software Multi Gauge (Fujifilm). When it is necessary, immunoblots were washed in PBS containing 1% NP-40 and 0.1% SDS instead of 0.1% PBST.

In the case of immunoblotting for pTyr, the same blot probed for pTyr was stripped and then re-probed with anti-FLAG antibody to confirm that equal amounts of the immunoprecipitated protein were loaded between control and drug-treated groups.

### Immunocytochemistry and confocal imaging

For surface immunostaining of NCKX2, live cells were incubated with rabbit anti-FLAG (1:2000) in serum-free Neurobasal A medium for 30 min at 4°C, rinsed with culture medium, fixed with ice-cold 4% PFA in PBS for 10 min and washed with PBS. For double-immunostaining with MAP2, these cells were subsequently permeabilized with 0.1% PBST for 5 min at RT, incubated in blocking solution (5% donkey serum in 0.1% PBST) for 1 h at RT, and then with anti-MAP2 (1:400, Millipore/Chemicon) diluted in blocking solution for 1h at RT (Bel et al., 2009). After three washes in PBS for 10 min, cells were incubated with Alexa Fluor 488-conjugated donkey anti-rabbit (1:100, Invitrogen/Molecular Probes) and Alexa Fluor 647-conjugated donkey anti-mouse (1:200, Jackson ImmunoResearch) diluted in blocking solution for 1 h at RT. Finally, the cells were washed three times in PBS and mounted with fluorescent mounting medium (DakoCytomation, Cambridge, UK).

For immunostaining of the internalized NCKX2, live cells were incubated with rabbit anti-FLAG (1:2000) in serum-free culture medium for 30 min at 37°C. After a brief wash with culture medium, cells were washed with acidic buffer (Neurobasal A medium, pH 2 with HCl) for 2 min to remove surface-bound antibody (Kurisu et al., 2010). Then cells were rinsed with PBS, fixed with ice-cold 4% PFA in PBS for 10 min and washed with PBS. Subsequent steps were the same as those for surface protein immunostaining.

The immunostained cells were imaged with FV300 (Olympus, Tokyo, Japan) or TCS-SP2 (Leica, Wetzlar, Germany) confocal laser scanning microscopes with 60× or 63× water-immersion objectives, and then processed using Fluoview or Leica Lite.

### Determination of somatodendritic and axonal fluorescence of NCKX2

To determine the fluorescence ratio of axonal to somatodendritic compartment (A/SD ratio) in a neuron, we made a binary mask image of a dendrite or an axon using the “ImageThreshold” routine of Igor Pro. A Tomato mask and a MAP2 mask were made from a tdTomato image and a MAP2 image, respectively. We defined the overlapping area of the Tomato mask (ROI of a transfected cell) and the MAP2 mask (ROIs of all somatodendritic neurites) as the somatodendritic ROI (SD-ROI) (Figure 2B). Then the axonal ROI was obtained by subtracting SD-ROI from the Tomato mask. Finally, the A/SD ratio was calculated by dividing the spatially averaged immunofluorescence intensity of NCKX2 over the axonal ROI by that of NCKX2 over the SD-ROI.

To determine the axonal or somatodendritic polarization for the subcellular distribution of surface NCKX2, we defined “normalized A/SD ratio” as an A/SD ratio of a protein normalized to that of tdTomato, which is assumed to display non-polarized subcellular distribution, in the same cell. If the normalized A/SD ratio of a protein is unity, we regarded the distribution of the protein as non-polarized. A value greater or less than one was regarded as preferential localization to the axon or to the somatodendritic region, respectively.

### Estimation of dendritic Ca^2+^ clearance in the hippocampal granule cells

Acute hippocampal slices (thickness, 300 μm) were prepared from P14–19 Sprague-Dawley rats as described in Lee et al. (Lee et al., 2007a). The whole-cell recordings were made at the soma of a hippocampal granule cell (GC) at 31–35°C. The K^+^-based pipette solution contained (in mM) 125 K-gluconate, 20 KCl, 20 HEPES, 5 Na-phosphocreatine, 4 MgATP, 0.3 Na_2_GTP, and 0.1 fura-4F (Invitrogen/Molecular probes) with pH adjusted at 7.3 using KOH. For a K^+^-free pipette solution (denoted as TMA^+^-based pipette solution), K-gluconate and KCl were replaced with equimolar tetramethylammonium (TMA)-gluconate and tetraethylammonium (TEA)-Cl, respectively. The artificial cerebrospinal fluid (ACSF) was composed of (in mM) 124 NaCl, 26 NaHCO_3_, 3.2 KCl, 2.5 CaCl_2_, 1.3 MgCl_2_, 1.25 NaH_2_PO_4_ and 10 glucose with pH adjusted at 7.4 by saturating with carbogen (95% O_2_, 5% CO_2_). For low [Na^+^] ACSF, 124 mM NaCl was replaced with equimolar choline-Cl. Serial images of fura-4F fluorescence were taken using a monochromator (Polychrome-IV, TILL Photonics, Martinsried, Germany) and an air-cooled slow-scan CCD camera (SensiCam; PCO, Kehlheim, Germany). We used the built-in on-chip binning (8 × 16 pixels) function to accelerate the frame rate (40 Hz; exposure time, 5 ms). In the off-line analysis, an ROI including the proximal dendrite was drawn on the fluorescence image of a GC, and the ratio (*R* = *F*_iso_/*F*_380_) of averaged fluorescence over the ROI at the isosbestic wavelength (360 nm; *F*_iso_) to that at 380 nm (*F*_380_) was converted to [Ca^2+^]_i_ according to the equation: [Ca^2+^]_i_ = *K*_eff_ · (*R* − *R*_min_) / (*R*_max_ − *R*), where *K*_eff_ was estimated as 8.12 μM. Calibration parameters were determined by “in-cell” calibration as described previously (Lee et al., 2008).

To quantify Ca^2+^ clearance, we analyzed the decay phase of a Ca^2+^ transient (CaT) evoked by a short depolarizing pulse under different conditions. Because Ca^2+^ clearance depends on the peak Δ[Ca^2+^] level, the duration of depolarization was adjusted in the range between 50 and 100 ms, such that the peak Δ[Ca^2+^] level of the evoked CaT is typically 1 μM. The decay phase was fitted with a biexponential function: *A*_0_ + *A*_1_ · *exp*(−*r*_1_ · *t*) + *A*_2_ · *exp*(−*r*_2_ · *t*). Assuming that intracellular Ca^2+^ buffers are alike among GCs, the weighted average of the rate constants (*r*_w_), which is defined as (*A*_1_ · *r*_1_ + *A*_2_ · *r*_2_)/(*A*_1_ + *A*_2_), can be regarded as a parameter representing Ca^2+^ clearance at the peak of the CaT, and exhibits little dependence on the peak Δ[Ca^2+^] level when it is higher than 0.8 μM (Lee et al., 2007b, 2009).

The SFK-activating peptide, EPQ(pY)EEIPIA, was synthesized by a commercial facility (AnyGen, Gwangju, South Korea). Unless otherwise specified, all small molecular weight drugs were purchased from Sigma (St. Louis, MO, USA).

### Statistical analysis

Data were analyzed using Igor Pro (version 6.2, WaveMetrics, Lake Oswego, OR, USA), and are presented as mean ± SEM. The statistical significance of differences between two experimental conditions was evaluated using Student's *t*-test using a significance level (*p*) of 0.05 or 0.01. That between more than two groups was evaluated using One-Way ANOVA. In Results, the first and the second statistical values in parentheses intervened by “vs.” represent statistical data under control conditions and under test conditions, respectively.

## Results

### NCKX2 interacts with the μ subunit of AP-2

**Figure 1:**
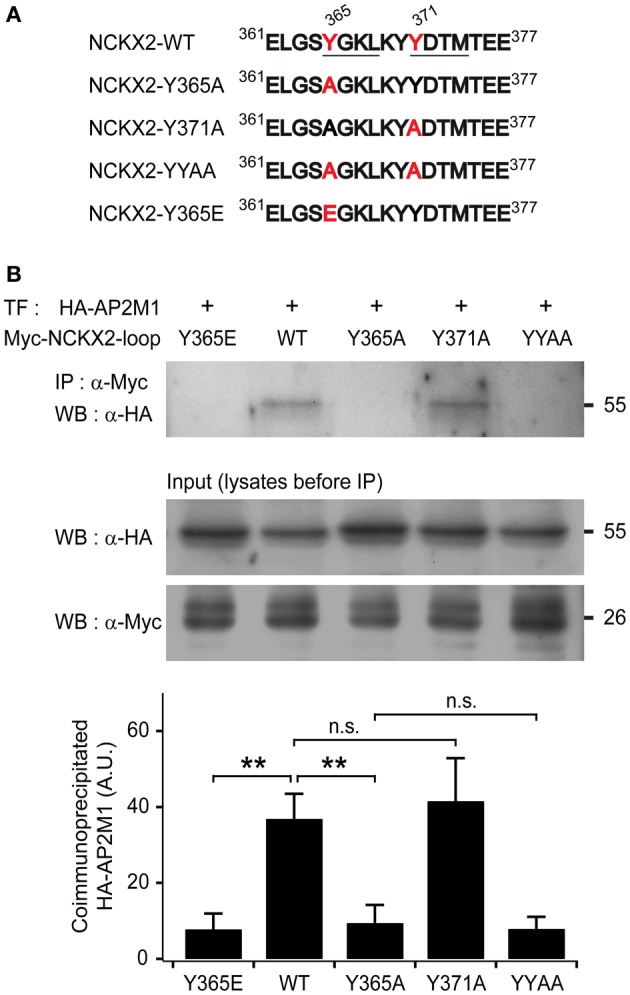
**The μ subunit of adaptor-binding protein 2 (AP2M1) interacts with NCKX2 but not with the Y365A mutant of NCKX2. (A)** The amino acid sequences of a part of cytoplasmic region of NCKX2. Two putative consensus tyrosine motifs (YxxΦ) that may interact with AP-2 are underlined (YGKL, aa 365-368; YDTM, aa 371-374). Amino acids (Tyr-365 and Tyr-371) mutated to alanine or glutamate are labeled in red. **(B)** The HA-tagged AP2M1 was coimmunoprecipitated with the wild-type or Y371A mutant of cytoplasmic loop of NCKX2 (NCKX2-loop), but not with any of the Y365A mutant, double mutant of Y365A and Y371A (YYAA), and the phosphomimetic mutant, Y365E (upper strip), when they were heterologously expressed in HEK293 cells. NCKX2 variants were tagged with c-myc. The loaded amounts of HA-AP2M1 and the different myc-NCKX2-loop variants in each of the transfected cell lysates before coimmunoprecipitation are shown in lower two strips. The lower bar graph shows the summary for the amount of coimmunoprecipitated HA-AP2M1 with each of the myc-NCKX2-loop variants (*n* = 5). Mean ± SEM; ^**^p < 0.01; n.s., not significant.

We have previously reported that dynamin-1-mediated endocytosis is involved in the low surface expression of NCKX2 in the somatodendritic region (Lee et al., 2012). Dynamin-1 is one of the key players in clathrin-mediated endocytosis. Given that clathrin-mediated endocytosis is responsible for the internalization of surface NCKX2, NCKX2 may interact with adaptor protein(s) of clathrin-coated vesicles. Clathrin-mediated endocytosis is initiated by recruitment of AP-2 to a target molecule. The cytosolic loop region of NCKX2 [NCKX2-loop, amino acids (aa) 288–478] has two tyrosine motifs (^365^YGKL and ^371^YDTM; Figure 1A), potential recognition sites for AP2M1.

To test whether NCKX2-loop interacts with AP2M1, c-myc-tagged NCKX2-loop (myc-NCKX2-loop) and HA-tagged AP2M1 (HA-AP2M1) were co-transfected into HEK293 cells. The cell lysate was immunoprecipitated with anti-c-myc IgG-conjugated agarose, and then HA-AP2M1 was detected by immunoblotting. We found that myc-NCKX2-loop was coimmunoprecipitated with HA-AP2M1 (Figure 1B).

### Tyr-365 of NCKX2 is necessary for interaction with AP2M1

To determine whether either or both of the two tyrosine motifs are important for the interaction, we created mutant constructs of myc-NCKX2-loop, in which the 365th tyrosine (Tyr-365) or the 371st tyrosine residue (Tyr-371) or both of them are mutated to alanine (Figure 1A; denoted as Y365A, Y371A, and YYAA, respectively). Using coimmunoprecipitation assay, we examined the interaction of HA-AP2M1 with each mutant that were expressed in HEK293 cells. Coimmunoprecipitation of HA-AP2M1 was significantly reduced by mutation of Y365A, but not by Y371A (Figure 1B; WT, 36.91 ± 6.57; Y365A, 9.46 ± 4.73, *p* < 0.01; Y371A, 41.60 ± 11.28, *p* = 0.73, *n* = 5). The amount of coimmunoprecipitated HA-AP2M1 was not further reduced by the mutation of YYAA compared with Y365A (Figure 1B; YYAA, 7.92 ± 3.12, *n* = 5, *p* = 0.79). These results indicate that Tyr-365, but not Tyr-371, of NCKX2 is necessary for interaction with AP2M1. To test whether phosphorylation of Tyr-365 is necessary for the interaction, we replaced Tyr-365 with glutamate (Figure 1A; denoted as Y365E). HA-AP2M1 was not coimmunoprecipitated with the Y365E mutant, suggesting that AP2M1 interacts with NCKX2 via dephosphorylated form of ^365^YGKL (Figure 1B; Y365E, 7.83 ± 4.12, *n* = 5, *p* < 0.01).

### The Y365A mutant of NCKX2 displays higher surface expression in the somatodendritic region than wild-type NCKX2

Given that NCKX2-Y365A is little recognized by AP2M1, the endocytosis of NCKX2-Y365A might be suppressed in somatodendritic regions of a neuron. To test this prediction, we transfected with FLAG-tagged WT or Y365A mutant of NCKX2 (NCKX2-WT or NCKX2-Y365A) in primary cultured rat hippocampal neurons and detected the surface and internalized NCKX2 using anti-FLAG antibody. For immunostaining of surface NCKX2, live cells were incubated with anti-FLAG at 4°C, a temperature prohibiting endocytosis. For immunolabeling of internalized NCKX2, live cells were first incubated with anti-FLAG at 37°C, an endocytosis-permissive temperature, and then surface-bound antibodies were removed by washing with an acidic buffer (pH 2). To visualize the entire morphology of the transfected neuron, a red fluorescent protein, tdTomato, was co-expressed.

**Figure 2:**
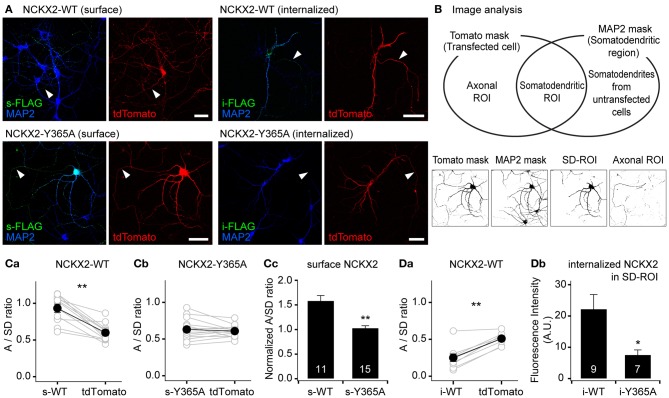
**The Y365A mutant of NCKX2 displays higher surface expression in the somatodendritic region than wild-type (WT) NCKX2. (A)** Immunocytochemical localization of surface or internalized FLAG-tagged NCKX2 in cultured hippocampal neurons. NCKX2-WT or NCKX2-Y365A were transfected at DIV7-8. To visualize the entire cell morphology, red fluorescent protein tdTomato (red) was cotransfected. At DIV12-14, hippocampal neurons were immunostained for FLAG (green) and a dendritic marker, MAP2 (blue). The surface NCKX2 was detected by the live-cell immunolabeling at 4°C. The internalized NCKX2 was immunolabeled by incubating live cells with anti-FLAG at 37°C, and then by removal of surface-bound antibody using an acidic medium. Arrowheads indicate axons. Scale bar: 50 μm. **(B)** Estimation of axon-to-somatodendrite ratio (A/SD ratio) of NCKX2 immunofluorescence. The Venn diagram depicts how the axonal- or somatodendritic (SD)-ROIs were defined. The representative binary masks were shown on the lower panels (the same cell as **A**). The overlapping area of tdTomato and MAP2 masks was defined as the SD-ROI. Then the axonal ROI was obtained by subtracting SD-ROI from tdTomato mask. **(C,D)** Summary of the mean A/SD ratios of surface or internalized NCKX2. **(Ca,b)** The A/SD ratio estimated from the immunofluorescence of surface NCKX2-WT **(Ca)**, but not of surface NCKX2-365A **(Cb)**, was significantly higher than that from tdTomato. **(Cc)** Each A/SD ratio of surface NCKX2 was normalized to that of tdTomato in the same cell. **(Da)** The A/SD ratio of the internalized NCKX2-WT was significantly lower than that of tdTomato. **(Db)** The immunofluorescence of the internalized NCKX2 in the somatodendritic compartment was significantly reduced by the Y365A mutation. Mean ± SEM; ^**^p < 0.01; ^*^p < 0.05.

Consistent with our previous studies (Lee et al., 2012), the surface expression of FLAG-tagged NCKX2-WT was not co-localized with the neurites immunoreactive for MAP2, a dendritic marker, indicating the polarized distribution of surface NCKX2 to the axonal region. In contrast, surface NCKX2-Y365A was distributed in the somatodendritic as well as in the axonal compartment (Figure 2A). On the other hand, the internalized NCKX2-WT was detected in both somatodendritic and axonal regions, whereas little immunoreactivity from internalized NCKX2-Y365A was detected (Figure 2A).

To quantify the polarized expression of wild type or Y365A mutant of NCKX2, we estimated the axon-to-somatodendrite ratio (A/SD ratio) from the averaged fluorescence intensities of surface or internalized NCKX2 over the axonal- and SD-ROIs (see “Materials and Methods”; Figure 2B). The A/SD ratio of surface NCKX2-WT (s-WT) was significantly higher than that of tdTomato in the same cell (Figure 2Ca; s-WT, 0.933 ± 0.056; tdTomato, 0.600 ± 0.037, *n* = 11, *p* < 0.01). In contrast, the surface expression of Y365A mutant of NCKX2 (s-Y365A) displayed no axonal polarization (Figure 2Cb; s-Y365A, 0.630 ± 0.038; tdTomato, 0.607 ± 0.020, *n* = 14, *p* = 0.62). Although tdTomato is assumed to display no polarized distribution in a neuron, the A/SD ratio of tdTomato was smaller than unity, most likely because the larger intracellular volume of the somatodendritic compartment is much larger than that of the axon (Rivera et al., 2003; Lewis et al., 2009). We normalized the A/SD ratio of NCKX2 to that of tdTomato, which was approximately 0.6 (denoted as “normalized A/SD ratio”). The normalized A/SD ratio of s-WT was higher than unity, indicating that s-WT is polarized to the axon (Figure 2Cc; 1.59 ± 0.10, *n* = 11). In contrast, the normalized A/SD ratio of s-Y365A was significantly lower than that of s-WT and close to unity (Figure 2Cc; 1.03 ± 0.04, *n* = 14, *p* < 0.01), indicating that the surface expression of NCKX2-Y365A is not polarized.

Next, we estimated the A/SD ratio of the immunofluorescence from internalized NCKX2-WT (i-WT) or NCKX2-Y365A (i-Y365A). Both the somatodendritic region and the axonal region were immunoreactive for i-WT, but immunofluorescence was much higher in the former than in the latter (Figure 2A). Consistently, the A/SD ratio of i-WT was significantly lower than that of tdTomato in the same cell (Figure 2Da; i-WT, 0.249 ± 0.052; tdTomato, 0.509 ± 0.026, *n* = 9, *p* < 0.01), indicating the internalization of NCKX2-WT is much stronger from the somatodendritic surface than the axonal surface. Because the immunoreactivity of i-Y365A was negligible in both axonal and somatodendritic regions (Figure 2A), it is meaningless to estimate the A/SD ratio of i-Y365A. When measured under the same imaging settings, the spatially-averaged immunofluorescence intensity of i-Y365A was significantly lower than that of i-WT in the somatodendritic compartment (Figure 2Db; i-WT, 22.3 ± 4.7, *n* = 9; i-Y365A, 7.6 ± 1.6, *n* = 7, *p* < 0.05), indicating that the Y365A mutation of NCKX2 prevents its somatodendritic endocytosis. These results strongly suggest that the Tyr-365 of NCKX2 is essential not only for the interaction with AP2M1 but also for the endocytosis of NCKX2 from the somatodendritic surface.

### Knockdown of AP2M1 abolishes the endocytosis of NCKX2

**Figure 3:**
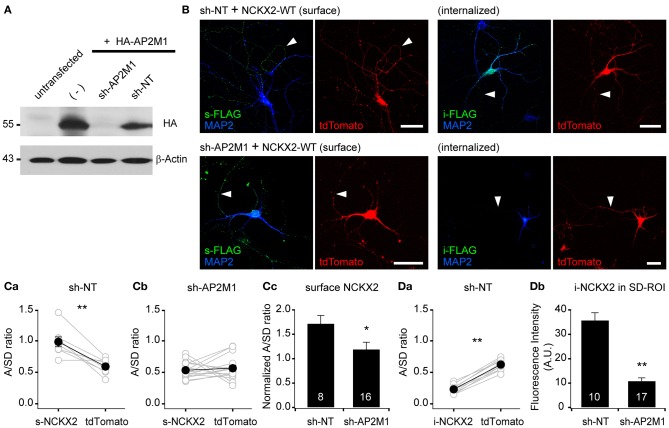
**Knockdown of AP2M1 abolishes the endocytosis of NCKX2. (A)** shRNA-mediated depletion of AP2M1. AP2M1-targeting shRNA (sh-AP2M1) was cotransfected with HA-tagged AP2M1 (HA-AP2M1) into HEK293 cells. sh-AP2M1 completely depleted HA-AP2M1, but non-targeting control shRNA (sh-NT) did not. The blot of β-actin is shown as a loading control. **(B)** FLAG-tagged wild-type NCKX2 (NCKX2-WT) was cotransfected with sh-AP2M1 or sh-NT into DIV8 hippocampal neurons. At DIV13-14, hippocampal neurons were immunostained for FLAG (green). Note that sh-AP2M1, but not sh-NT, caused a dramatic reduction of internalized NCKX2-WT and an increase in the surface expression of NCKX2-WT in the somatodendritic region. Arrowheads indicate axons. Scale bar: 50 μm. **(C,D)** Summary for the mean A/SD ratios of surface or internalized NCKX2-WT. **(Ca,b)** The A/SD ratio of surface NCKX2-WT was significantly higher than that of tdTomato in the sh-NT expressing neurons (**Ca**; s-NCKX2, 0.983 ± 0.007; tdTomato, 0.591 ± 0.042, *n* = 8, *p* < 0.01), but not different in the AP2M1-depleted neurons (**Cb**; s-NCKX2, 0.534 ± 0.033; tdTomato, 0.565 ± 0.047, *n* = 16, *p* = 0.59). **(Cc)** The tdTomato-normalized A/SD ratio of surface NCKX2 was significantly lower in the sh-AP2M1 expressing neurons than sh-NT expressing one (sh-NT, 1.722 ± 0.163, *n* = 8; sh-AP2M1, 1.195 ± 0.142, *n* = 16, *p* < 0.05). **(Da)** The A/SD ratio of the internalized NCKX2-WT was significantly lower than that of tdTomato (i-NCKX2, 0.231 ± 0.022; tdTomato, 0.623 ± 0.028, *n* = 10, *p* < 0.01). **(Db)** The spatially averaged immunofluorescence of internalized NCKX2-WT on SD-ROI was significantly reduced in the AP2M1-depleted neurons (sh-NT, 35.8 ± 3.0, *n* = 10; sh-AP2M1, 10.9 ± 1.3, *n* = 17, *p* < 0.01). Mean ± SEM; ^**^p < 0.01; ^*^p < 0.05.

To verify the contribution of AP2M1 to the endocytosis of NCKX2, we studied the effect of shRNA-mediated depletion of AP2M1 on the NCKX2 trafficking. The knockdown efficiency of AP2M1-targeting shRNA (sh-AP2M1) was tested in HEK293 cells heterologously expressing HA-AP2M1 together with sh-AP2M1 or non-targeting control shRNA (sh-NT). The sh-AP2M1, but not the sh-NT, completely depleted HA-AP2M1 (Figure 3A). Next, we tested whether the depletion of AP2M1 abolishes the endocytosis of NCKX2. FLAG-tagged NCKX2-WT was cotransfected with sh-AP2M1 and tdTomato (as a morphological marker) into DIV8 hippocampal neurons. Five or six days later, surface or internalized NCKX2 was detected by immunolabeling with anti-FLAG antibody. Compared with the neurons expressing sh-NT, those expressing sh-AP2M1 displayed higher surface expression of NCKX2 in the somatodendritic region and a dramatic reduction of internalized NCKX2 (Figures 3B–D). As is evident from the normalized A/SD ratio in Figure 3C, sh-AP2M1 abolished axonal polarization of surface NCKX2. These results suggest that the surface expression of NCKX2 is maintained low by AP2M1-mediated endocytosis in somatodendritic compartments.

### Src family kinase-mediated Tyr-365 phosphorylation of NCKX2 regulates its surface expression

The inhibitory effect of the phosphomimetic mutation at Tyr-365 (Y365E) on the interaction with AP2M1 raises a possibility that endocytosis of NCKX2 could be regulated by phosphorylation of Tyr-365 (Figure 1B). Endocytosis of many neuronal membrane proteins, such as the β3 and γ 2 subunits of the GABA_A_ receptor (Kittler et al., 2005, 2008; Jurd et al., 2010) and the GluN2B subunit of the NMDA receptor (Prybylowski et al., 2005), is regulated by tyrosine phosphorylation of the tyrosine (YxxΦ) motif by a SFK. In general, phosphorylation of a tyrosine motif by SFK inhibits its interaction with AP2M1 and subsequent endocytosis, resulting in the enhancement of surface expression levels. We examined the possibility that phosphorylation of Tyr-365 in the tyrosine motif (YGKL) of NCKX2 by SFK may prevent its endocytosis to increase the surface expression of NCKX2.

**Figure 4:**
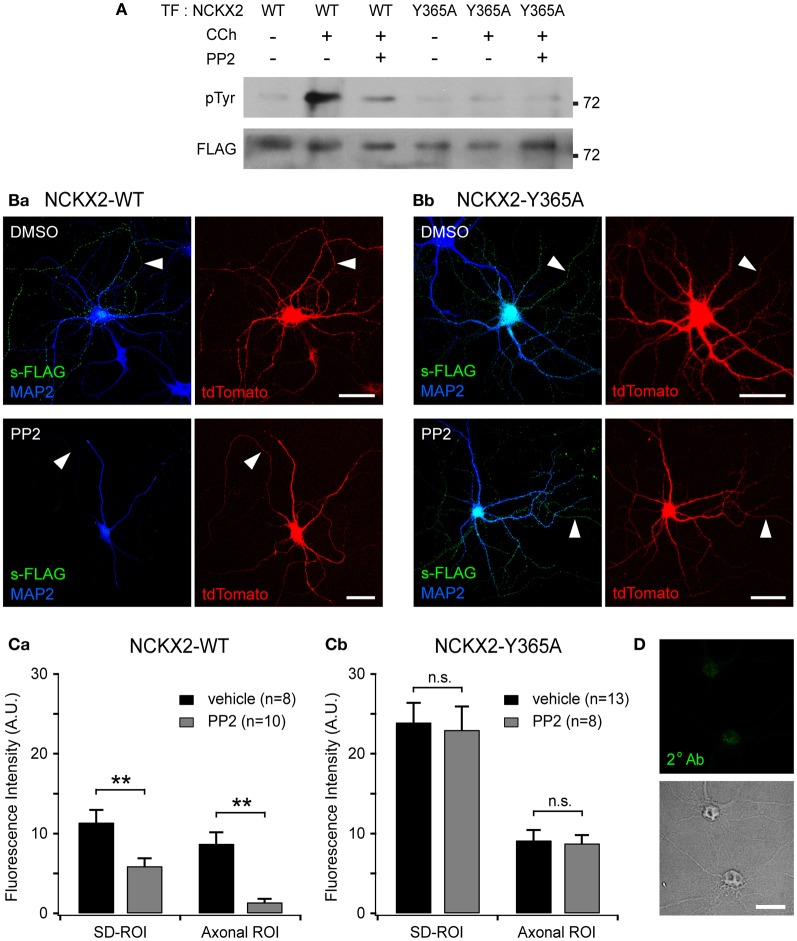
**Src family kinase (SFK)-mediated Tyr-365 phosphorylation of NCKX2 regulates its surface expression. (A)** Tyrosine phosphorylation of NCKX2 in response to carbachol (CCh) in PC-12 cells. We transfected FLAG-tagged NCKX2-WT or NCKX2-Y365A into PC-12 cells which normally express PYK2 and SFK, and treated the cells with 1 mM CCh for 2 min. To observe tyrosine-phosphorylation of NCKX2, NCKX2 was immunoprecipitated with anti-FLAG and then immunoblotted with anti-phosphotyrosine (pTyr) IgG. NCKX2-WT, but not NCKX2-Y365A, was strongly phosphorylated by CCh treatment. Pretreatment with 10 μM PP2, a selective inhibitor of SFK, reduced the CCh-induced tyrosine phosphorylation of NCKX2-WT. The FLAG signals on the same blot are shown in the lower image, showing little difference in expression levels of wild-type or Y365A mutant NCKX2 between different conditions. **(B)** Hippocampal neurons expressing FLAG-tagged NCKX2-WT **(Ba)** or NCKX2-Y365A **(Bb)** were treated with 0.1% DMSO (control; upper) or 10 μM PP2 (bottom) for 2 h. Wild-type or Y365A mutant of NCKX2 expressed on the surface was visualized with anti-FLAG (s-FLAG; green). Dendrites were immunostained with anti-MAP2 (blue). Arrowheads indicate axons. Scale bar: 50 μm. **(C)** The spatially averaged fluorescence intensity of wild-type **(Ca)** or Y365A mutant **(Cb)** of surface NCKX2 on SD- or axonal ROIs of the vehicle (DMSO; black)- or PP2 (gray)-treated neurons under the same imaging settings. ^**^p < 0.01; n.s., not significant. **(D)** DIV8 hippocampal neurons were incubated with the secondary antibody (Alexa Fluor 488-conjugated donkey anti-rabbit IgG) without prior immunolabeling with a primary antibody. In the permeabilized condition, the somata (most likely nuclei, shown in the transmitted image) display non-specific staining. Scale bar: 20 μm.

First, we tested whether SFK can phosphorylate the tyrosine motif of NCKX2. One of the upstream signaling molecules of SFK is proline-rich tyrosine kinase2 (PYK2), which is activated by intracellular Ca^2+^ elevation or PKC (Lev et al., 1995). Once Ca^2+^ triggers the phosphorylation of PYK2, activation of PYK2 and SFKs is maintained by reciprocal tyrosine phosphorylation (Girault et al., 1999). It is well known that PYK2 and SFK can be activated by CCh in PC-12 cells (Lev et al., 1995; Dikic et al., 1996). We transfected FLAG-tagged NCKX2-WT or NCKX2-Y365A into PC-12 cells, which normally express PYK2 and SFK, and treated the cells with 1 mM CCh for 2 min to activate PYK2 (Lev et al., 1995). To detect phosphorylated NCKX2, NCKX2 was immunoprecipitated with anti-FLAG-conjugated agarose beads, and then immunoblotted with anti-phosphotyrosine (pTyr) IgG. CCh significantly increased the tyrosine-phosphorylation of NCKX2-WT but not of NCKX2-Y365A (Figure 4A), implying that Tyr-365 is the major target residue of the PYK2-SFK signaling cascade. Furthermore, the CCh-induced phosphorylation of Tyr-365 in NCKX2-WT was inhibited by pretreatment with PP2 (10 μM, for 30 min), a selective inhibitor of SFK (Figure 4A), indicating that SFK is involved in the CCh-induced tyrosine-phosphorylation of NCKX2. We confirmed that there was little difference in expression levels of WT or Y365A mutant NCKX2 between control and CCh-treated groups by detection of FLAG in the same blot (Figure 4A).

Given that the tyrosine motif of NCKX2 can be phosphorylated by SFK, we next tested whether the surface expression of NCKX2 can be reduced by PP2. We transfected FLAG-tagged NCKX2-WT or NCKX2-Y365A into primary cultured hippocampal neurons at DIV8, and treated the neurons at DIV12 with PP2 (10 μM) or vehicle (0.1% DMSO) for 2 h. Observing surface NCKX2 by live-cell immunolabeling with anti-FLAG IgG, we found that PP2, but not vehicle, reduced the expression level of NCKX2-WT both on the axonal and somatodendritic surfaces (Figure 4Ba). To quantify the surface expression level of NCKX2, spatially-averaged immunofluorescence intensity of NCKX2 was measured in the SD- and axonal ROIs. As described above, SD- or axonal ROIs were defined based on the region immunoreactive for MAP2 and the tdTomato-positive region (Figure 2B). The A/SD ratio of surface NCKX2-WT in the vehicle-treated control neurons was not different from that of non-treated controls in Figure 2Ca [0.79 ± 0.12 (*n* = 8) and 0.93 ± 0.06 (*n* = 11), respectively; *p* = 0.25]. The inhibition of SFK by PP2 caused a decrease in the immunoreactivity of surface NCKX2 on both the somatodendritic and axonal surfaces [Figure 4Ca; SD-ROI, 11.41 ± 1.55 (*n* = 8) vs. 5.92 ± 0.97 (*n* = 10); Axonal ROI, 8.74 ± 1.43 (*n* = 8) vs. 1.40 ± 0.42 (*n* = 10), *p* < 0.01]. These results indicate that Tyr-365 of NCKX2 can be phosphorylated by SFK and that SFK-dependent tyrosine phosphorylation of NCKX2 is essential for the somatodendritic and axonal surface expression. Since the Y365A mutant is not recognized by AP2M1 (Figure 1B), it is expected that surface expression of the Y365A mutant is not affected by PP2. We found that PP2 did not suppress the surface expression of Y365A mutant on the somatodendritic compartments [Figures 4Bb,Cb; SD-ROI, 23.93 ± 2.44 (*n* = 13) vs. 23.01 ± 2.93 (*n* = 8), *p* = 0.81; Axonal ROI, 9.16 ± 1.27 (*n* = 13) vs. 8.79 ± 1.01 (*n* = 8), *p* = 0.84], confirming that the inhibitory effect of PP2 on surface expression of NCKX2 results from inhibition of tyrosine phosphorylation of Tyr-365 residue of NCKX2. Note that neurons were permeabilized to double-immunolabel for MAP2 after live-cell immunolabeling of FLAG. The remaining green immunofluorescence in the somatic region after treatment with PP2 may be caused by nonspecific staining of the nucleus by the secondary antibody, because fluorophore-conjugated secondary antibody stained nuclei without a primary antibody under the permeabilized conditions (Figure 4D). This non-specific immunolabeling may contribute to the higher immunofluorescence in the SD-ROI than in the axonal ROI in the presence of PP2 (Figure 4Ca).

### Activation of Src family kinase enhances the NCKX activity in the proximal dendrite

**Figure 5:**
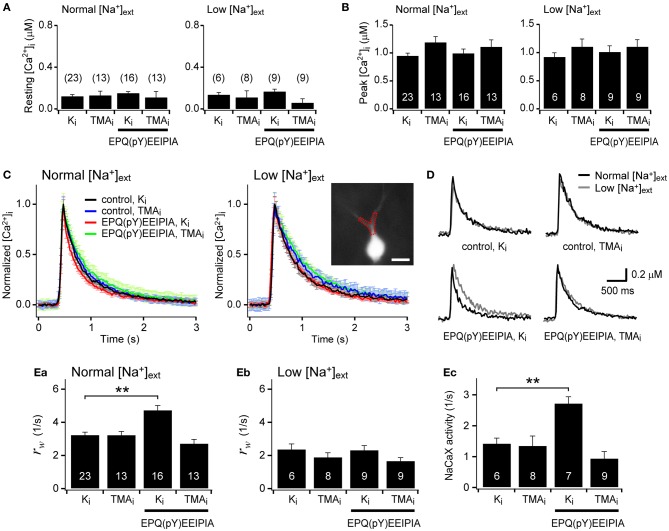
**Activation of Src family kinase (SFK) enhances the NCKX activity in the proximal dendrite of dentate granule cells. (A,B)** Resting and peak [Ca^2+^]_i_ levels were not significantly different between experimental conditions. **(C)** Averaged traces of the normalized Ca^2+^ transients (CaTs) evoked by a short depolarizing pulse under normal or low extracellular Na^+^ concentrations ([Na^+^]_ext_). Each panel shows four overlaid averaged traces under different conditions. K_i_ and TMA_i_ denote K^+^- and TMA^+^-based pipette solutions, respectively. For red and green traces, 1 mM EPQ(pY)EEIPIA, a SFK-activating peptide, was included in the pipette solution. EPQ(pY)EEIPIA significantly accelerated the Ca^2+^ decay rate only under K^+^-based internal and normal [Na^+^]_ext_ conditions (red lines). Error bars shown in light colors represent SEM. *Inset*, A fura-4F fluorescence image of a granule cell. A typical proximal dendritic ROI is shown as a red dotted polygon. Scale bar: 20 μm. **(D)** Effects of low [Na^+^]_ext_ on the Ca^2+^ decay rate under different conditions. Each panel shows overlaid representative CaTs recorded under the indicated intracellular condition (black, normal [Na^+^]_ext_; gray, low [Na^+^]_ext_). **(E)** Summary for Ca^2+^ clearance and Na^+^/Ca^2+^ exchange (NaCaX) activity. Ca^2+^ clearance was quantified as a weighted average of rate constants (*r*_*w*_) estimated from the biexponential fit to the decay phase of individual CaTs **(Ea,b)**. The difference in the *r*_*w*_ values between normal and low [Na^+^]_ext_ conditions in the same cell was regarded as the NaCaX activity **(Ec)**. Mean ± SEM; ^**^p < 0.01.

We inquired whether the activation of SFKs can enhance the dendritic NCKX activity. To address this question, we estimated Ca^2+^ clearance in proximal dendrites of hippocampal GCs before and after SFK activation. To activate SFK, we included in the patch pipette a SFK-activating peptide [EPQ(pY)EEIPIA], which disrupts intramolecular interaction between the SH2 domain and phosphorylated tail of SFK (Liu et al., 1993). There was no significant difference in the resting [Ca^2+^]_i_ level between eight experimental conditions [*F*_(7, 89)_ = 0.751, *p* = 0.630 by One-Way ANOVA; Figure 5A]. Ca^2+^ clearance was estimated from analysis of the decay phase of Ca^2+^ transients (CaTs) evoked by a short depolarizing pulse (−70 to 0 mV) in the GCs loaded with 100 μM fura-4F, a Ca^2+^ indicator dye (*K*_D_ ~0.77 μM), via a whole-cell patch pipette. The pulse duration (50–100 ms) was adjusted such that the peak of the CaTs was between 0.8 and 1.2 μM, and thus the peak values were not different between eight experimental conditions [*F*_(7, 89)_ = 1.10, *p* = 0.370; Figure 5B]. We compared Ca^2+^ clearance under eight different conditions: normal or low extracellular [Na^+^] ([Na^+^]_ext_); K^+^- or TMA^+^-based pipette solution (denoted as K_i_ or TMA_i_, respectively); with or without 1 mM EPQ(pY)EEIPIA in the pipette solution. Averaged traces of CaTs that were normalized to the peak in each condition are superimposed in Figure 5C. Representative traces of CaTs measured in the same cell under normal and low [Na^+^]_ext_ conditions are compared in each panel of Figure 5D under four different conditions. Intracellular perfusion of EPQ(pY)EEIPIA accelerated the Ca^2+^ decay rate under normal [Na^+^]_ext_ and K_i_ conditions but not under low [Na^+^]_ext_ or TMA_i_ conditions (Figures 5C,D). We fitted a biexponential function to the decay phase of an individual CaT, and regarded the weighted average of rate constants (*r*_*w*_) of the fitted function as a parameter for Ca^2+^ clearance (Lee et al., 2007b). Figures 5Ea,Eb show the summary for Ca^2+^ clearance under four different internal conditions in normal and low [Na^+^]_ext_, respectively. Under normal [Na^+^]_ext_ conditions, Ca^2+^ clearance estimated as *r*_*w*_ was significantly accelerated by SFK-activating peptide only under K^+^-internal conditions [3.26 ± 0.15 (*n* = 23) vs. 4.76 ± 0.26 (*n* = 16), *p* < 0.01], and was not different between other three conditions [*F*_(2, 46)_ = 2.363, *p* = 0.105; Figure 5Ea]. Under low [Na^±^]_ext_ conditions, Ca^2±^ clearance was not different between the four experimental conditions [*F*_(3, 28)_ = 2.422, *p* = 0.087; Figure 5Eb]. We estimated the Na^+^/Ca^2+^ exchanger (NaCaX) activity in a GC as a difference between the *r*_*w*_ value measured in the normal [Na^+^]_ext_ and that in the low [Na^+^]_ext_ (Figure 5Ec). Consistent with our previous reports (Lee et al., 2009), the dendritic NaCaX activity was little altered by TMA^+^-based pipette solution, indicating that most NaCaX activity can be attributed to NCX rather than NCKX (Figure 5Ec; K_i_, 1.44 ± 0.16/s, *n* = 6; TMA_i_, 1.36 ± 0.31/s, *n* = 8, *p* = 0.85). In contrast, application of EPQ(pY)EEIPIA greatly enhanced the NaCaX activity under the K_i_ condition but not under the TMA_i_ condition (Figure 5Ec; K_i_, 2.74 ± 0.20/s, *n* = 7, *p* < 0.01; TMA_i_, 0.95 ± 0.21/s, *n* = 9, *p* = 0.28). These results suggest that NCKX rather than NCX is responsible for the NaCaX activity enhanced by SFK activation in the proximal dendrite. Therefore, we conclude that the surface expression of NCKX is regulated by its endocytosis, and the endocytosis is regulated by SFK-dependent phosphorylation of Tyr-365.

## Discussion

NCKX plays a pivotal role in calcium clearance in the axon terminal but little contributes to that in the somatodendritic region (Lee et al., 2002, 2009; Kim et al., 2003). Consistently, we have previously reported that the surface expression of NCKX2 is polarized to the axon, and that the axonal polarization of NCKX2 is maintained by two mechanisms (Lee et al., 2012). One is axonal transport of NCKX2 via KIF21A, and the other is selective endocytosis of NCKX2 in the somatodendritic compartment. Supporting this view, the present study shows that the selective endocytosis is mediated by interaction of the tyrosine motif in the loop region of NCKX2 with AP2M1. Furthermore, we demonstrated that SFK-dependent tyrosine phosphorylation regulates the endocytosis of NCKX2 and thus its surface expression in the somatodendritic region.

### Role of SFK-dependent regulation of NCKX2 activity in local Ca^2+^-homeostasis

Calcium homeostasis is crucial not only for the cell survival but also for normal Ca^2+^ signaling. Neurons are multiply compartmentalized, and they display uneven subcellular distributions of neurotransmitter receptors and ion channels that can strongly influence local Ca^2+^ influx. Local Ca^2+^ homeostasis is a prerequisite for normal Ca^2+^ signaling in a given compartment. Although Ca^2+^ itself can regulate the gene transcription of calcium channels and transporters (Carafoli et al., 1999), the regulation of such gene transcription may not be sufficient for local Ca^2+^ homeostasis. The cellular mechanism by which a neuron balances the local input and output of calcium is little understood.

SFKs are implicated in long-term potentiation and associated trafficking of many excitatory neurotransmitter receptors including GluA2, GluN1, GluN2A, and GluN2B (Grosshans et al., 2002; Ohnishi et al., 2011). SFK-dependent tyrosine phosphorylation enhances the surface expression of these receptors. Given that these receptors mediate depolarization or Ca^2+^ influx, activation of SFK may potentially perturb Ca^2+^ homeostasis without being counter-balanced by a corresponding readjustment of CCMs.

**Figure 6:**
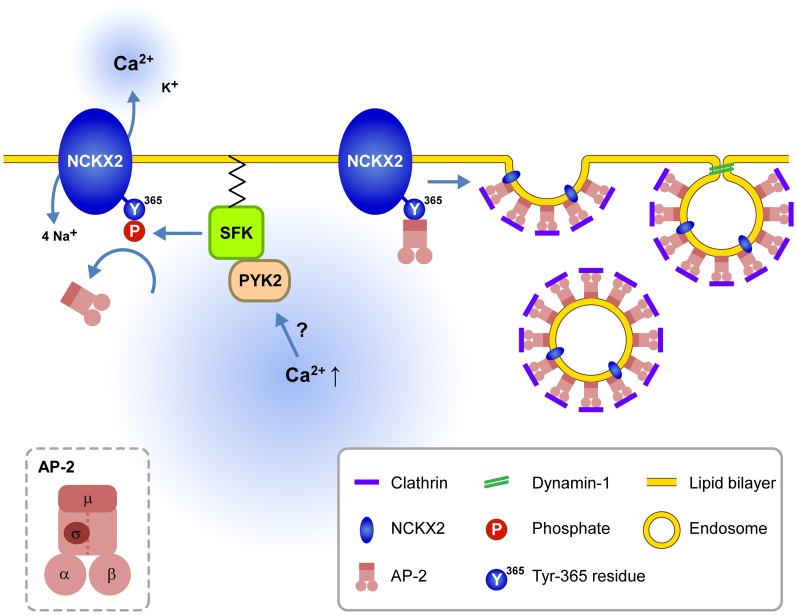
**Schematic illustration for the signaling pathways regulating endocytosis of NCKX2 in the somatodendritic compartment.** Under resting conditions, the unphosphorylated or dephosphorylated tyrosine motif of NCKX2 interacts with AP-2 and thereby NCKX2 undergoes endocytosis. When elevation of cytosolic [Ca^2+^] activates PYK2-SFK signaling pathway, activated SFK phosphorylates Tyr-365 of NCKX2, which in turn prevents interaction of AP-2 with NCKX2. Without interaction with AP2M1, NCKX2 avoids endocytosis resulting in higher surface expression of NCKX2 and enhanced Ca^2+^ clearance. These processes may contribute to local Ca^2+^ homeostasis. *Inset*, the subunit structure of AP-2.

The present study suggests that dendritic NCKX2 activity may undergo dynamic modulation depending on the dendritic SFK activity. Supporting this view, the CCh-induced SFK activation of a short period (2 min) is enough to phosphorylate the Tyr-365 residue of NCKX2 expressed in PC-12 cells (Figure 4A). Furthermore, we observed that the dendritic NCKX activity was enhanced within typically 10 min after a break-in when the hippocampal GC was intracellularly perfused with SFK-activating peptide via a whole-cell patch pipette (Figure 5). PYK2 and PKC have been implicated as upstream signaling molecules of SFK in neurons (Lu et al., 1999). It is notable that Ca^2+^ increase itself can trigger activation of PYK2 (Lev et al., 1995; Huang et al., 2001), raising a possibility that cytosolic elevation of Ca^2+^ may trigger the surface expression of NCKX2 in the somatodendritic region. Given that SFK is a downstream effector of a Ca^2+^-dependent kinases, our results may provide a possible mechanism for local Ca^2+^ homeostasis in a central neuron (Figure 6).

The other implication of our study is the role of alternative splicing of NCKX2. The tyrosine motifs of NCKX2 are located in the region that can be removed by alternative splicing (Tsoi et al., 1998). Our results imply that such a spliced isoform may be resistant to endocytosis, and thus more readily surface-expressed in somatodendritic regions than the unspliced isoform. Therefore, the somatodendritic expression of NCKX2 may be regulated not only by tyrosine phosphorylation but also by the expression level of spliced NCKX2. Recently, NCKX2 has been implicated to play a protective role in ischemic brain damage (Cuomo et al., 2008). It remains to be elucidated whether brain ischemia and consequent elevation of resting cytosolic [Ca^2+^] can enhance the surface expression of NCKX2 in the somatodendritic regions of central neurons.

### Re-interpretation of previous studies in light of our results

When the NCKX activity was estimated from calcium clearance during decay phases of CaTs, NCKX little contributed to calcium clearance in the somatodendritic compartment (Kim et al., 2003; Lee et al., 2009). In contrast, reverse mode NCKX activity was clearly observed in the soma of forebrain neurons (Kiedrowski et al., 2004; Cuomo et al., 2008). In the former studies, the somatic NCKX activity may be underestimated because NCKX activity was estimated from the decrease of calcium clearance caused by inhibition of NCKX, which may induce compensatory activation of other CCMs (Kim et al., 2003; Lee et al., 2009). Furthermore, the discrepancy may be caused by unphysiological experimental conditions used for measurement of the reverse mode NCKX activity in the latter studies. The reverse mode NCKX activity causes the cytosolic [Ca^2+^] to rise to the tens of micromolar range (Kiedrowski, 2004). In light of the present study, such high cytosolic [Ca^2+^] may enhance the surface expression of NCKX2 in the somatodendritic region through Ca^2+^-dependent activation of PYK2 and subsequent tyrosine phosphorylation of NCKX2 (Figure 6).

Previously, we have reported that a PKC activator, phorbol 12, 13-dibutyrate (PDBu), enhances the NCKX activity at the calyx of Held (Lee et al., 2006). This finding was interpreted as PKC-dependent direct phosphorylation of NCKX2, because the activity of NCKX2 heterologously expressed in HEK293 cells was also enhanced by PDBu (Lee et al., 2006). PKC is one of the upstream molecules that activate SFK (Lu et al., 1999). In light of the present study, the PDBu-induced increase in the NCKX2 activity is necessary to be re-evaluated, as HEK293 cells weakly express endogenous Src kinases (Holmes et al., 1996). Accordingly, further studies need to test whether activation of SFK is involved in the PDBu-induced increase in the NCKX activity at the calyx of Held. PKC may activate NCKX2 not only directly but also through a PYK2-SFK signaling pathway. The present study provides a possibility that the latter can enhance the NCKX activity by an increase in the surface expression of NCKX2 at the calyx of Held.

### Possible molecular mechanisms underlying differential endocytosis of NCKX2 between somatodendritic and axonal compartments

The effects of dynamin inhibitors (Lee et al., 2012) and sh-AP2M1 (Figure 3) indicate that NCKX2 is continuously eliminated from the somatodendritic surface by the mechanism of endocytosis under control conditions. Axonal polarization of Na_v_1.2, VAMP2 and Caspr2 is similarly dependent on preferential somatodendritic removal by endocytosis (selective retention hypothesis) (Garrido et al., 2001; Sampo et al., 2003; Bel et al., 2009). In case of NgCAM, somatodendritic endocytosis not only suppresses the somatodendritic surface expression but also is coupled to its axonal transport (dendrite-to-axon transcytosis of somatodendritic endosome). Thus, the transcytosis of NgCAM depends on its cytosolic domain which contains canonical AP-2 recognition motif (Wisco et al., 2003). It remains to be answered whether the transcytosis mechanism is involved in the axonal polarization of NCKX2. The axonal polarization of NCKX2, however, might not entirely depend on the transcytosis mechanism, because the axonal surface expression of NCKX2 was not abolished by inhibition of endocytosis in this and our previous studies (Lee et al., 2012).

The molecular mechanism for selective endocytosis is not well understood. PKC-dependent constitutive phosphorylation of scaffold-binding region in Caspr2 is required for somatodendritic selective endocytosis (Bel et al., 2009). Interaction of Na_v_1.2 with an axonal binding partner such as ankyrin G prevents endocytosis from axonal membrane, resulting in selective endocytosis from somatodendritic compartments (Leterrier et al., 2010). Recently, myosin VI has been proposed to be involved in differential endocytic rate of NgCAM between two compartments (Lewis et al., 2011).

As inhibition of SFK using PP2 reduced the surface expression of NCKX2 not only in the soma and dendrites but also in axonal regions (Figure 4), phosphorylation of the tyrosine motif might be a primary determinant for the surface expression of NCKX2 in both compartments. Since NCKX2 can be surface-expressed under conditions in which somatodendritic SFK is activated (Figure 5), the lower surface expression of NCKX2 in the somatodendritic region suggests that most NCKX2 molecules in this compartment are not tyrosine-phosphorylated under the resting conditions. These results imply that axonal NCKX2 may be more tyrosine-phosphorylated than somatodendritic NCKX2, and that differential phosphorylation of the tyrosine motif underlies the differential endocytosis in two compartments. To our knowledge, however, there is no previous example showing that axonally polarized surface expression of a molecule is maintained by differential phosphorylation of a tyrosine motif in the molecule. It is little understood, however, whether SFKs are constitutively active in axonal regions. If tyrosine-phosphorylated NCKX2 is polarized to the axon under resting conditions, it needs to be further studied whether it is associated with differences between somatodendritic and axonal regions in SFK activity and/or counteracting molecules such as tyrosine phosphatases.

### Conflict of interest statement

The authors declare that the research was conducted in the absence of any commercial or financial relationships that could be construed as a potential conflict of interest.
